# Induction of *A Disintegrin and Metalloproteinase with Thrombospondin motifs 1* by a rare variant or cognitive activities reduces hippocampal amyloid-β and consequent Alzheimer’s disease risk

**DOI:** 10.3389/fnagi.2022.896522

**Published:** 2022-08-09

**Authors:** Yunjie Qiu, Longze Sha, Xiuneng Zhang, Guanjun Li, Wanwan Zhu, Qi Xu

**Affiliations:** ^1^State Key Laboratory of Medical Molecular Biology, School of Basic Medicine Peking Union Medical College, Institute of Basic Medical Sciences Chinese Academy of Medical Sciences, Beijing, China; ^2^Neuroscience Center, Chinese Academy of Medical Sciences, Beijing, China

**Keywords:** *ADAMTS1*, genetic variant, cognitive activities, amyloid precursor protein, amyloid-β, Alzheimer’s disease

## Abstract

Amyloid-β (Aβ) derived from amyloid precursor protein (APP) hydrolysis is acknowledged as the predominant hallmark of Alzheimer’s disease (AD) that especially correlates to genetics and daily activities. In 2019, meta-analysis of AD has discovered five new risk loci among which *A Disintegrin and Metalloproteinase with Thrombospondin motifs 1* (*ADAMTS1*) has been further suggested in 2021 and 2022. To verify the association, we re-sequenced *ADAMTS1* of clinical AD samples and subsequently identified a novel rare variant c.–2067A > C with watchable relevance (whereas the *P*-value was not significant after adjustment). Dual-luciferase assay showed that the variant sharply stimulated *ADAMTS1* expression. In addition, *ADAMTS1* was also clearly induced by pentylenetetrazol-ignited neuronal activity and enriched environment (EE). Inspired by the above findings, we investigated ADAMTS1’s role in APP metabolism *in vitro* and *in vivo*. Results showed that ADAMTS1 participated in APP hydrolysis and consequently decreased Aβ generation through inhibiting β-secretase-mediated cleavage. In addition, we also verified that the hippocampal amyloid load of AD mouse model was alleviated by the introduction of *ADAMTS1*, and thus spatial cognition was restored as well. This study revealed the contribution of *ADAMTS1* to the connection of genetic and acquired factors with APP metabolism, and its potential in reducing hippocampal amyloid and consequent risk of AD.

## Introduction

Alzheimer’s disease (AD) is a progressive disorder characterized by cerebral amyloid-β (Aβ) deposition as an early pathological alteration (Jack et al., 2018). Currently, the unbalanced processing of Aβ, finally leading to the formation of amyloid plaques in the brain, is considered to be the initiating event of AD as suggested by available evidence (Jack et al., 2010). This process occurs when individuals are still cognitively normal, as demonstrated by numerous follow-up cohort studies in which Aβ positivity is reached many years before dementia onset (Hanseeuw et al., 2019). The initial Aβ processing may be affected by multiple factors that hereby influence the risk of AD, of which genetics or daily activities may keep individuals retardant or progressive to the amyloid plaque during early life.

Due to the significance of genetic factors, recent studies have focused on identifying associated genes of AD. In 2019, the genome-wide association study implicated 25 risk loci, and positive signals were found in or near genes that encoded proteins participating in amyloid precursor protein (APP) metabolism. Further analysis showed that the up-mentioned variants were associated not only with the early onset autosomal dominant familial AD but also the late-onset type (Kunkle et al., 2019). Moreover, one newly identified locus, *A Disintegrin and Metalloproteinase with Thrombospondin motifs 1* (*ADAMTS1*), has been further suggested in the meta-analysis of AD in 2021 and 2022 (Schwartzentruber et al., 2021; Bellenguez et al., 2022). *ADAMTS1* encodes a zinc metalloprotease belonging to the ADAMTS secretase family (Lemarchant et al., 2013) that functions on extracellular matrix (ECM) hydrolysis (Zhong and Khalil, 2019). ADAMTSs are activated and secreted to hydrolyze substrates, including proteoglycans, collagen, and laminin (Porter et al., 2005). Although ADAMTSs have been studied as hydrolases for decades, the function of the specific member ADAMTS1 in nervous system is still poorly understood. Regarding the adjacency of *ADAMTS1* to *APP* and the role of APP as an ECM component (Beher et al., 1996; Caceres and Brandan, 1997; van der Kant and Goldstein, 2015), we have proposed that ADAMTS1 acts on APP processing and thereby contributes to Aβ generation.

In addition to genetics, cognitive activities may also affect the onset and progression of AD. Numerous longitudinal studies have investigated the relation between cognitive activities and AD progression, suggesting that cognitive enrichment may account for less cognitive decline and AD pathology in old age (Vemuri et al., 2014; Oveisgharan et al., 2020). Moreover, some studies have implied that mice participating in cognitive activities exhibit elevated ECM hydrolase (Murase et al., 2019; Nguyen et al., 2020). Thus, hypothesis that cognitive activities may stimulate associated ECM hydrolase and subsequently function on APP processing ties cognitive enrichment to less AD pathology. However, more evidence is still needed to support the hypothesis, and it is significant to understand the molecular connections of activities to APP processing to reduce the risk of AD.

According to the up-mentioned background, we checked the association of *ADAMTS1* with AD and studied whether *ADAMTS1* was induced by cognitive activities and involved in APP metabolism. To this end, we performed gene re-sequencing using clinical samples, tested the role of neural activities in the induction of *ADAMTS1*, and explored the mechanism of its function on Aβ pathology *in vitro* and *in vivo*. The results demonstrated that a newly identified genetic variant in promoter and cognitive activities both induced *ADAMTS1* expression, which stimulated APP’s conversion to a novel product and thus decreased Aβ generation. Our findings identified ADAMTS1 as a novel APP hydrolase that connected the genetics and cognitive activities with hippocampal amyloid processing.

## Materials and methods

### Clinical samples

Human blood was collected from AD patients (1,152 cases) and control subjects (864 cases) who had given written consent to this study approved by the Institutional Review Board of Chinese Academy of Medical Sciences and Peking Union Medical College (CAMS and PUMC, Beijing, China). Genomic DNA was extracted using Quickgene DNA whole blood kit (DB-L, KURABO, Osaka, Japan) following the manufacturer’s instructions.

### Re-sequencing

11 pairs of primers were used to amplify the promoter and exon region of *ADAMTS1*, and the PCR products were then subjected to Sanger sequencing. The sequencing data were compared with records of NCBI to search for potential single nucleotide polymorphisms (SNPs). Primers used are shown in Table 1.

**TABLE 1:** Primers used in the re-sequencing test.

Region	Primers
Promoter	F: 5′-GTACGGATGGCTTTGCCTTCAAGC-3′
	R: 5′-GTCTCTTGGTTGGCTCCAAGTAG-3′
	F: 5′-CAAGTAAGCAATCTCGCTAGG-3′
	R: 5′-AGCCTGCCAGGAGCTCCTTAG-3′
	F: 5′-AGTCGTCTCTGGTGAAGAGGTG-3′
	R: 5′-CTGAGGCAACGCGGAGATTGGT-3′
Exon 1	F: 5′-TAACAATCCAGAGCAGGCCAACG-3′
	R: 5′-TCTGTCCGCGGACTTATGATCCT-3′
Exon 2	F: 5′-CAGAAGAAATCCTGCTCACACAC-3′
	R: 5′-AGGGTGTGCCTTCACATATG-3′
Exon 3	F: 5′-ATGGTACCAGCTGTGAGACT-3′
	R: 5′-GGATGATATGCAGCAGGTTC-3′
Exon 4	F: 5′-GAGATGGAGTCTCACTCTGTCT-3′
	R: 5′-GACAGGAAGTTATTGATCTGCG-3′
Exon 5	F: 5′-CGCAGATCAATAACTTCCTGTC-3′
	R: 5′-ACCGCACGTTCTCGAACAGTCT-3′
Exon 6/7	F: 5′-GGTGTATCAACGGCAAGTGTGT-3′
	R: 5′-GCAGGCAGTTGCCAATTAATG-3′
Exon 8	F: 5′-CTTAGCTGGAGGAGACATAGGT-3′
	R: 5′-ACTTCGATGTTGGTGGCTCCAGT-3′
Exon 9	F: 5′-AATGGGCCTTTCAACAGGTCTG-3′
	R: 5′-CACCTTACTGATACACCTCACTGG-3′

### Dual-luciferase reporter assay

HEK 293T cells were co-transfected with pGL3-Basic (firefly luciferase plasmid) carrying required sequences and pRL-TK (Renilla luciferase plasmid). After 48-h culture, reporter luciferase activity was measured and normalized to Renilla luciferase using Dual-Luciferase Reporter Assay System (E1910, Promega, Madison, United States).

### Animals and drug administration

Wild-type (WT) adult male C57BL/6J mice (7–8 weeks old) were obtained from SPF Biotechnology Co., Ltd. (Beijing, China) and raised together for 2–3 weeks to adapt to the environment before experiments. 5 × FAD lines were maintained by crossing WT mice (C57BL/6J background) with 5 × FAD mice (B6SJL background, #034840-JAX, The Jackson Laboratory, Bar Harbor, United States). Only male mice were used and non-transgenic WT littermate mice served as control. WT and 5 × FAD mice infected with recombinant adeno-associated virus (rAAV)2/9-*hSyn*-*EGFP* [empty vector (Mock)] or rAAV2/9-*hSyn*-*ADAMTS1*-*FLAG* in hippocampus were, respectively, named as WT-Mock, WT-ADAMTS1 (ATS1), 5 × FAD-Mock and 5 × FAD-ATS1. Animals were housed under standard conditions of 22°C and 12-h light/dark cycle with free access to water and food. Animal care and handling were performed according to terms approved by the Institutional Review Board of CAMS and PUMC (Beijing, China).

Drugs were intraperitoneally injected and dose was 55 mg/kg for pentylenetetrazol (PTZ, P6500, Sigma-Aldrich, Shanghai, China) according to Matsu-ura’s work with little modification (Matsu-ura et al., 2002) and 400 mg/kg for chloral hydrate (Zemdegs et al., 2019; Cheng et al., 2021) (A600288, Sangon Biotech, Shanghai, China). Each group contained four mice for this analysis.

### Enriched environment

Mice in Enriched environment (EE) were housed for 24 h in rat cages measuring 30 cm W × 18 cm H × 46 cm L. Standard nesting, rodent foraging toys, and one running wheel were arranged in the cage. Additionally, metal link chains or small wooden blocks were suspended from the cage roof. Mice housed in normal cages without decorations were considered as control. Each group contained four mice for this analysis.

### Immunohistochemistry

Mice deeply anesthetized by pentobarbital sodium (50 mg/kg) were transcardially perfused with PBS. Brains were quickly harvested and fixed in 4% paraformaldehyde for 24 h at 4°C. Then, the tissue was embedded in paraffin and made into 4-μm sections which were deparaffinized by gradient ethanol and subjected to antigen retrieve in citrate buffer.

Sections for Aβ dyeing were blocked by 5% BSA for 1 h at room temperature (RT) and incubated with primary antibodies overnight at 4°C. Next, after three-time washing using PBS buffer, sections were incubated with fluorescent secondary antibodies for 2 h at RT. After another three-time washing using PBS buffer, sections were photographed through upright microscopy (DM6 B, Leica Microsystems, Shanghai, China). Images of 5 × magnification in hippocampus were captured on three sections per animal. Aβ load was evaluated by Image J software 1.52. After a fixed intensity threshold was set using Image J, measurements were performed for positive area covered by Aβ staining on each image. The numbers of animals used for this analysis were seven for 5 × FAD-Mock and six for 5 × FAD-ATS1.

Sections for ADAMTS1 staining were incubated in 3% H_2_O_2_ for 30 min at RT followed by blocking in 5% BSA for 1 h at RT and incubation with primary antibody overnight at 4°C. Next, after washing using PBS buffer, sections were incubated with HRP-conjugated secondary antibody that reacted with tyramine-coupled fluorescence (B40943, Thermo Fisher Scientific, Waltham, United States). Next, antigen retrieval was performed again to label NeuN, GFAP or iba1. The rest steps were the same as above described.

Antibodies were diluted with PBS buffer containing 1% BSA and listed below: anti-β-amyloid antibody (1:800, RRID:AB_2565328, 803015, BioLegend, San Diego, United States), anti-ADAMTS1 antibody (1:100, RRID:AB_2877879, 12749-1-AP, Proteintech, Wuhan, China), anti-NeuN antibody (1:500, RRID:AB_2880708, 26975-1-AP, Proteintech, Wuhan, China), anti-GFAP antibody (1:500, RRID:AB_2631098, 12389, Cell Signaling Technology, Danvers, United States), and anti-iba1 antibody (1:200, RRID:AB_2832244, ab178847, Abcam, Cambridge, United Kingdom).

### Quantitative real-time polymerase chain reaction

Hippocampal RNA was extracted using TRIzol reagent (15596026, Thermo Fisher Scientific, Waltham, United States) following the manufacturer’s protocol. cDNA was then prepared using Transcriptor First Strand cDNA Synthesis Kit (04379012001, Roche, Basel, Switzerland) following the manufacturer’s protocol and stored at –20°C until use. Quantitative real-time polymerase chain reaction (qPCR) was performed using real-time PCR System (CFX Connect, Bio-Rad, Hercules, United States). Transcription levels of *Adamts1*, *Fos*, *Npas4*, and *Zif268* were tested using FastStart Essential DNA Green Master mix (06402712001, Roche, Basel, Switzerland). The fold change of mRNA was normalized to *Gapdh* using the 2^–ΔΔ*Ct*^ method. Primer sequences are shown in Table 2.

**TABLE 2:** Primers used in qPCR experiments.

Gene	Primers
*Adamts1*	F: 5′-TGAATGGTGTGAGTGGCGAT-3′
	R: 5′-CCATCAAACATTCCCCGTGT-3′
*Arc*	F: 5′-GCCAGTCTTGGGCAGCATAG-3′
	R: 5′-GTATGAATCACTGCTGGGGGC-3′
*Fos*	F: 5′-CGGCAGAAGGGGCAAAGTAG-3′
	R: 5′-AGTTGATCTGTCTCCGCTTGG-3′
*Npas4*	F: 5′-CTCTGGATGCTGATCGCCTT-3′
	R: 5′-CAGGTGGGTGAGCATGGAAT-3′
*Zif268*	F: 5′-TATGAGCACCTGACCACAGAGTC-3′
	R: 5′-TAGGTGATGGGAGGCAACCG-3′
*Gapdh*	F: 5′-CGACTTCAACAGCAACTCCCACTCTTC-3′
	R: 5′-TGGGTGGTCCAGGGTTTCTTACTCCTT-3′

### Plasmids construction and transfection

The cDNAs of *ADAMTS1*, *human APP Swedish mutation (swAPP)*, *ADAMTS1*-*FLAG*, *APP*-*MYC*, *ARC*, *FOS*, *NPAS4* and *ZIF268* were synthesized by Tsingke Biotechnology Co., Ltd. (Beijing, China) and cloned into the pcDNA3.1 vector. Mutations in *ADAMTS1* (namely, *E402A* and *E402Q*) were generated by overlap extension PCR method and then cloned into pcDNA3.1 vector. NEOFECT DNA-transfection reagent (TF201201, Neofect Biotech, Beijing, China) was used for the transient transfection of plasmids in accordance with the manufacturer’s instructions.

### Western blot assay

Cells washed with PBS buffer were lysed in CelLytic lysis buffer (C2978, Sigma-Aldrich, Shanghai, China) containing protease inhibitors. Whole proteins were obtained through centrifugation at 12,000 *g* for 15 min at 4°C. Hippocampi acquired from fresh brains were homogenized in 2% SDS buffer and centrifuged for 15 min at 12,000 *g* at 16°C to collect whole proteins. Protein concentration was determined by the bicinchoninic acid method after proper dilution. Then, the protein samples were denatured by mixing with 5 × sample loading buffer and a total of 20-μg protein of each sample was separated by SDS-PAGE and transferred to nitrocellulose membrane by the wet method. Specially, to detect C-terminal fragments of APP (CTFs), samples were mixed with 2 × sample loading buffer (P1325, Solarbio, Beijing, China), separated by Tris-tricine SDS-PAGE (P1320, Solarbio, Beijing, China) and finally transferred to PVDF membrane by the wet method. The membrane was then successively treated by blocking (in TBST buffer containing 5% skim milk for 1 h at RT), incubation with primary antibodies (in TBST buffer containing 5% skim milk overnight at 4°C), washing (three times in TBST buffer), incubation with secondary antibody (in TBST buffer containing 5% skim milk for 1 h at RT), repetitive washing (three times in TBST buffer) and finally developed using ECL reagents (WBULS0500, Millipore, Burlington, United States). The ratio of “target to β-actin” of treated groups was normalized to those of respective control groups and interpreted to fold change (i.e., relative level). Specially, for PTZ experiments, samples from different groups were loaded on the same one gel and thus four replications were separated into four membranes. For each replication, relative levels of different timepoints were normalized to that of PBS group which thus in each membrane was set to “1” without SEM (Suzuki et al., 2012; Sha et al., 2017).

Antibodies were diluted with TBST buffer containing 5% skim milk and listed below: anti-ADAMTS1 antibody for checking ADAMTS1 overexpression in cells and 5 × FAD mice (1:2,000, RRID:AB_11212782, MAB1810, Millipore, Burlington, United States), anti-ADAMTS1 antibody for checking endogenous ADAMTS1 expression in PTZ and EE experiments (1:2,000, ab276133, Abcam, Cambridge, United Kingdom), anti-Arc antibody (1:5,000, RRID:AB_2151832, 16290-1-AP, Proteintech, Wuhan, China), anti-c-fos antibody (1:2,000, RRID:AB_2247211, 2250, Cell Signaling Technology, Danvers, United States), anti-zif268 antibody (1:2,000, RRID:AB_11182923, 22008-1-AP, Proteintech, Wuhan, China), anti-APP antibody (1:20,000, RRID:AB_2289606, ab32136, Abcam, Cambridge, United Kingdom), anti-sAPP antibody (1:10,000, RRID:AB_94882, MAB348, Millipore, Burlington, United States), anti-sAPPα antibody (1:1,000, RRID:AB_1630819, JP11088, Immuno-Biological Laboratories, Männedorf, Switzerland), anti-sAPPβ-WT antibody (1:1,000, RRID:AB_1630824, JP18957, Immuno-Biological Laboratories, Männedorf, Switzerland), anti-sAPPβ-Swedish antibody (1:1,000, RRID:AB_1630822, JP10321, Immuno-Biological Laboratories, Männedorf, Switzerland), and anti-β-actin antibody (1:100,000, RRID:AB_2223172, 4970, Cell Signaling Technology, Danvers, United States).

### Enzyme-linked immunosorbent assay

Concentration of Aβ_40_ and Aβ_42_ was measured using enzyme-linked immunosorbent assay (ELISA) kits following the manufacturer’s protocol (KHB3481, KHB3441, Thermo Fisher Scientific, Waltham, United States). Tissue samples were prepared through homogenizing hippocampus in RIPA lysis buffer (R0020, Solarbio, Beijing, China) containing protease inhibitors and centrifugation at 16,000 *g* for 20 min at 4°C, while cell samples were the conditioned medium of 48-h cultured HEK 293T cells co-transfected with *swAPP* and *ADAMTS1* plasmids.

### Vector construction and recombinant adeno-associated virus packaging

rAAV was constructed through cloning *ADAMTS1*’s cDNA accompanied by either CMV promoter for primary neuron transfection or *Synapsin* promoter for animal experiments into AAV vector. Next, rAAV2/9-CMV*-ADAMTS1*-3 × *FLAG* and rAAV2/9-CMV-3 × *FLAG* were packaged by OBiO Technology Co., Ltd (Shanghai, China) while rAAV2/9*-hSyn-EGFP* and rAAV2/9*-hSyn-ADAMTS1-FLAG* were packaged by Taitool Bioscience Co., Ltd (Shanghai, China). The final titer of each rAAV was 3–5 × 10^12^ virus genome (V.G.)/mL.

### Primary neuronal cultures and chemical treatment

Primary neurons were from E15.5 embryos of ICR mice. Briefly, cerebral cortex was gently separated and digested by 1.5% trypsin at 37°C for 15 min. Suspended cells were harvested and seeded in six-well plates (1.2–1.5 × 10^6^ cells/well) pre-coated with poly-D-lysine. Cultures were maintained with Neurobasal Plus medium (A3582901, Thermo Fisher Scientific, Waltham, United States) containing B27 Plus supplement (A3582801, Thermo Fisher Scientific, Waltham, United States) at 37°C in a humidified 5% CO_2_ incubator, and half of the medium was replaced regularly. Transfection was performed using rAAV2/9-CMV*-ADAMTS1*-3 × *FLAG* or Mock on days *in vitro* (*DIV*) 7. Specifically, the virus suspension was diluted with Neurobasal Plus medium containing B27 Plus supplement and then 1 × 10^10^ V.G. in total with or without *ADAMTS1* was added into neuron culture; serial dilution of rAAV2/9-CMV*-ADAMTS1*-3 × *FLAG* was also conducted using Neurobasal Plus medium containing B27 Plus supplement and final virus amount for each group was 7.5, 5, 2.5, 1.25, and 0 × 10^9^ V.G. in total. Cells were cultured for another 10–12 days before use, and half of the medium was replaced regularly during this period.

Primary neurons on *DIV* 15–17 were treated with ADAM10 inhibitor GI254023X (HY-19956, MCE, New Jersey, United States) or BACE1 inhibitor verubecestat (HY-16759, MCE, New Jersey, United States) which were initially dissolved in DMSO to prepare the stock solution preserved at −20°C (5 mM for GI254023X and 1 mM for verubecestat). Final concentration was set at 20 μM for GI254023X (the final ratio of DMSO was 0.4%) and 1 μM for verubecestat (the final ratio of DMSO was 0.1%) by proper dilution with medium in the culture system. Extracellular medium and neurons were collected 2 d later.

### Cellular component separation

Components of HEK 293T cells were separated using cell fractionation kit (SM-005, Invent Biotechnologies, Plymouth, United Kingdom) following the manufacturer’s protocol into cytosol, organelles and plasma membrane which were then diluted to equal volume with non-denatured protein solubilization reagent (WA-010, Invent Biotechnologies, Plymouth, United Kingdom).

### Co-immunoprecipitation

HEK 293T cells co-transfected with *ADAMTS1-FLAG* and *APP-Myc* were washed with PBS buffer and solubilized in ice-bath by CelLytic lysis buffer (C2978, Sigma-Aldrich, Shanghai, China) containing protease inhibitors. Clear lysates were incubated with primary antibodies overnight at 4°C and then with protein G Agarose beads (11243233001, Roche, Basel, Switzerland) for 2 h at 4°C. After five-time rinse with TBS buffer, immunoprecipitated proteins were recovered from the beads by boiling for 5 min in sample buffer, and then analyzed by immunoblotting.

Antibodies were diluted with TBST buffer containing 5% skim milk and listed below: anti-FLAG tag antibody (1:5,000, RRID:AB_262044, F1804, Sigma-Aldrich, Burlington, United States), anti-Myc tag antibody (1:5,000, RRID:AB_331783, 2276, Cell Signaling Technology, Danvers, United States) and anti-Na^+^/K^+^ ATPase antibody (1:5,000, RRID:AB_2227873, 14418-1-AP, Proteintech, Wuhan, China).

### Brain stereotaxic injection

Four month-old male 5 × FAD mice were deeply anesthetized and immobilized on a stereotaxic frame. The coordinates of hippocampus were set as *X* ± 2.0 mm from bregma, *Y* –2.0 mm from bregma and *Z* –1.8 mm. 1.0 μL of rAAV suspension (4 × 10^9^ V.G. in total) carrying *ADAMTS1-FLAG* or Mock was then injected into both pieces of hippocampus at 0.2 μL/min and the expression of *ADAMTS1-FLAG* was controlled by *Synapsin* promoter. Syringe was slowly withdrawn 10 min later and mice were kindly housed for 2 months before other tests.

### Morris water maze test

Morris water maze was a round, water-filled tub (140 cm in diameter) equipped with cues and an invisible escape platform placed at a fixed spatial location. Mice were consecutively trained 20 times in total (four times per day) in the maze to locate the escape platform with the assistance of cues in 60 s at most, or else they would be manually guided. Their swimming routes and latency time (from release to landing) were recorded. Once getting on the platform, mice were rendered extra 15 s to explore surroundings. Following this training, a probe trial without the escape platform was conducted to record the time mice spent in the platform quadrant. ANYMAZE software 5.11 was utilized for data collection and further analysis. The numbers of animals used in this behavioral analysis were 14 for WT-Mock, 10 for WT-ATS1, 18 for 5 × FAD-Mock, and 14 for 5 × FAD-ATS1.

### Statistics

The odd ratios (ORs), 95% confidence intervals, and *P*-values of alleles were calculated by *chi*-squared test to evaluate the association of SNPs with AD. Data were shown as mean ± SEM. Difference was analyzed using GraphPad Prism 6. Unpaired Student’s *t*-test or Mann-Whitney *U*-test was used to analyze the significance between two experimental groups and one-way ANOVA or Kruskal-Wallis test was used for multiple comparisons depending on data distribution verified by skewness, kurtosis, and Shapiro-Wilk test (Kim, 2013; Alexander et al., 2017; Ellenberger et al., 2020). Specially, two-way ANOVA was used for animal behavior analysis (Qiu et al., 2007). It was considered as statistically significant when *P*-value was less than 0.05.

## Results

### A rare variant suggested by its weak association with lower Alzheimer’s disease risk stimulated *A Disintegrin and Metalloproteinase with Thrombospondin motifs 1* expression

**FIGURE 1:**
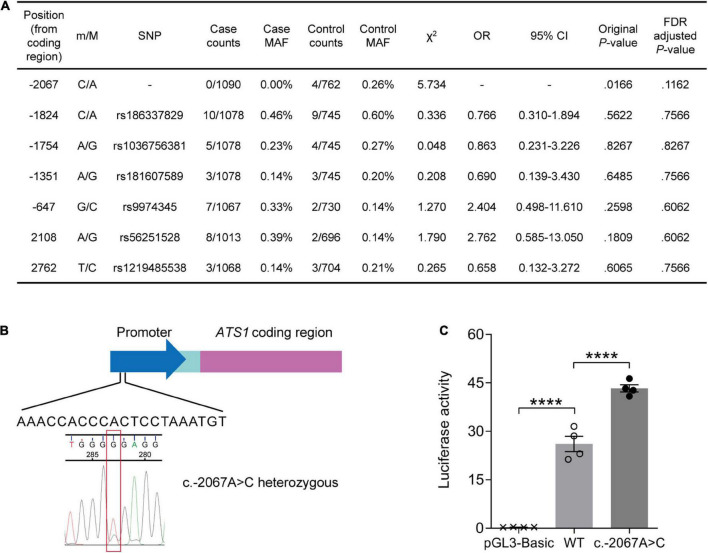
A rare variant suggested by its weak association with lower AD risk stimulated *ATS1* expression. **(A)** *ATS1* re-sequencing was conducted using clinical samples from AD patients and healthy aged individuals. Statistical results of SNPs were shown. **(B)** A schematic diagram showing the position of SNP c.–2067A > C in *ATS1*. **(C)** HEK 293T cells transfected with the *ATS1* WT or c.–2067A > C promoter were subjected to dual-luciferase assay. The relative luciferase activity indicated that c.–2067A > C promoted *ATS1* transcription compared with the WT promoter (*n* = 4, *P* < 0.0001, c.–2067A > C vs. WT, one-way ANOVA). ****P < 0.0001. Data were expressed as Mean ± SEM. AD, Alzheimer’s disease; ATS1, ADAMTS1; CI, confidence interval; FDR, false discovery rate; MAF, minor allele frequency; OR, odds ratio; SNP, single nucleotide polymorphism; WT, wild type.

We performed re-sequencing of promoter and exon regions to assess the association of *ADAMTS1* with AD. As a result, two missense mutations in the exon and five SNPs in the promoter were identified (Figure 1A). The SNP at –2067 where adenine was substituted with cytosine (c.–2067A > C, Figure 1B) was not reported previously. Statistical analysis suggested that the rare c.–2067A > C [case minor allele frequency (MAF) = 0.00%, control MAF = 0.26%] had a weak association with AD before adjustment (original *P* = 0.0166, false discovery rate-adjusted *P* = 0.1162), indicating a potential protective role of c.–2067A > C against AD.

Since the novel variant located within the promoter, dual-luciferase reporter assay was then performed to explore the effect of c.–2067A > C on gene expression. As illustrated in Figure 1C, enhanced luciferase activity was induced by c.–2067A > C (∼50%, *P* < 0.0001, compared with WT group). Thus, individuals carrying c.–2067A > C might have a high transcription level of *ADAMTS1*. Considering the potential negative association of c.-2067A > C with AD, it was suggested that the high-level ADAMTS1 might protect carriers to some extent against the risk of AD.

### Activity-related brain activities contributed to the induction of *A Disintegrin and Metalloproteinase with Thrombospondin motifs 1*

**FIGURE 2:**
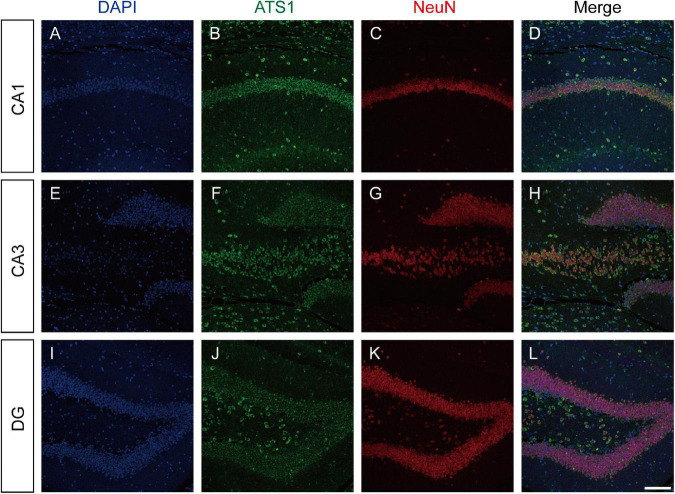
ATS1 was mainly expressed in neurons. **(A,E,I)** Nuclei were labeled by DAPI in CA1, CA3, and DG regions. **(B,F,J)** ATS1 was labeled by anti-ATS1 antibody in CA1, CA3, and DG regions. **(C,G,K)** Neurons were labeled by anti-NeuN antibody in CA1, CA3, and DG regions. **(D,H,L)** The merged images of DAPI, ATS1, NeuN revealed that neurons were the predominant source of ATS1. Scale bars, 100 μm. ATS1, ADAMTS1. DG, dentate gyrus.

The expression pattern of ADAMTS1 in WT mouse hippocampus was examined by labeling ADAMTS1 simultaneously with NeuN, GFAP or iba1. Results presented in Figure 2 and Supplementary Figure 1 showed that neurons dominated the expression in CA1, CA3 and dentate gyrus.

**FIGURE 3:**
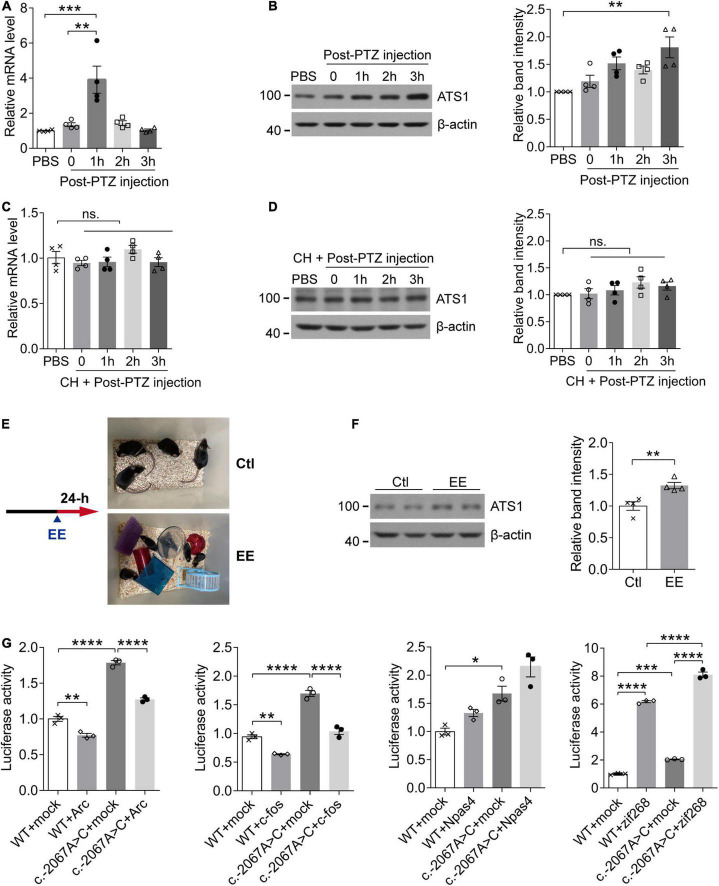
The activity-dependent expression pattern of *ATS1* through the potential mediation of zif268. **(A,B)** Ten week-old WT male mice were intraperitoneally injected with PTZ to induce synaptic activation and sacrificed at different timepoints. **(A)** The *Ats1* mRNA was significantly induced 1-h post-injection (*n* = 4, *P* < 0.001, 1 h vs. PBS; *P* < 0.01, 1 h vs. 0, one-way ANOVA). **(B)** Western blot of ATS1 at different timepoints. The relative band intensities showed that ATS1 was obviously upregulated 3-h post-injection (*n* = 4, *P* < 0.01, 3 h vs. PBS, Kruskal-Wallis test). **(C,D)** Ten week-old WT male mice were intraperitoneally injected with CH 30 min before PTZ administration and sacrificed at different timepoints. No significant difference was detected for *Ats1* **(C)** mRNA or **(D)** protein levels when compared with PBS group (*n* = 4, one-way ANOVA). **(E)** Scheme of EE experiments. **(F)** Western blot of ATS1 in hippocampus after 24-h EE-housing. The relative band intensities indicated upregulated expression of ATS1 (*n* = 4, *P* < 0.01, EE vs. Ctl, Student’s *t*-test). **(G)** HEK 293T cells transfected with the *ATS1* promoter (WT or c.–2067A > C) and immediate early gene plasmids *ARC*, *FOS*, *NPAS4*, or *ZIF268* were subjected to the dual-luciferase assay. The relative luciferase activity demonstrated that zif268 multiplied *ATS1* promoter activity significantly (*n* = 3, *P* < 0.0001, WT + zif268 vs. WT + mock; *P* < 0.0001, c.–2067A > C + zif268 vs. c.–2067A > C + mock; *P* < 0.0001, c.–2067A > C + zif268 vs. WT + zif268, one-way ANOVA). *P < 0.05, **P < 0.01, ***P < 0.001, ****P < 0.0001. Data were expressed as Mean ± SEM. ATS1, ADAMTS1; CH, chloral hydrate; ns., no significance; Ctl, control; EE, enriched environment; PTZ, pentylenetetrazol; WT, wild type; mock, empty vector.

In rodent models, neural activities trigger the elimination of ECM surrounding neurons (Nagy et al., 2006; Michaluk et al., 2009), and ADAMTS1, as an ECM hydrolase, acts on the degradation of proteoglycans that constitutes the matrix (Kuno et al., 2000). Considering these two aspects, we next investigated whether *ADAMTS1* expression was related to neuronal activity. PTZ was administrated to WT mice to induce neuronal activation in hippocampus, and subsequent qPCR and western blot revealed that *Adamts1* clearly increased at both mRNA (especially 1 h post injection, *P* < 0.001, compared with PBS group) and protein levels (especially 3 h post injection, *P* < 0.01, compared with PBS group) (Figures 3A,B). Furthermore, the PTZ-induced *Adamts1* upregulation was entirely suppressed by chloral hydrate pre-treatment (Figures 3C,D), verifying that the overexpression was triggered by the neuronal activation rather than other effects of PTZ. Additionally, mice raised in EE also held enhanced ADAMTS1 levels (shown in Figures 3E,F, *P* < 0.01, compared with control group). Taken together, the data suggested that the expression of *ADAMTS1* was related to brain activities.

The expression of immediate-early genes (IEGs) identifies neuronal ensembles activated during cognition encoding (Bozon et al., 2003; Plath et al., 2006; Kubik et al., 2007; Lin et al., 2008). Since *Adamts1* expression was regulated by neuronal activity, it was of interest to screen the transcription factor responsible for bridging *ADAMTS1* upregulation and activities. In the current work, several fast-reacting IEGs (zif268, c-fos, Arc and Npas4) were selected because *Adamts1* quickly responded 1-h post-injection. qPCR and western blot results revealed that these genes clearly increased in response to PTZ-induced neuronal activity (Supplementary Figures 2A,B), which had been suggested by previous studies as well (Retchkiman et al., 1996; Flood et al., 2004; Wang et al., 2007; Szyndler et al., 2013; Yun et al., 2013; Reschke et al., 2017; Wu et al., 2017; Shan et al., 2018). Next, *ZIF268*, *ARC*, *FOS*, and *NPAS4* were, respectively, introduced into luciferase system to investigate the managing role of IEGs on *ADAMTS1* expression, and zif268 was shown to have the dominated role (Figure 3G, *P* < 0.0001, compared with respective mock control). Meanwhile, the rare SNP c.-2067A > C identified by re-sequencing obviously enhanced the effects of zif268 on the promoter activity of *ADAMTS1* (Figure 3G, *P* < 0.0001, compared with WT + zif268). Therefore, the data suggested that the induction of *ADAMTS1* by neural activities might be ascribed to the mediation of IEG zif268.

### *A Disintegrin and Metalloproteinase with Thrombospondin motifs 1* was involved in amyloid precursor protein metabolism

**FIGURE 4:**
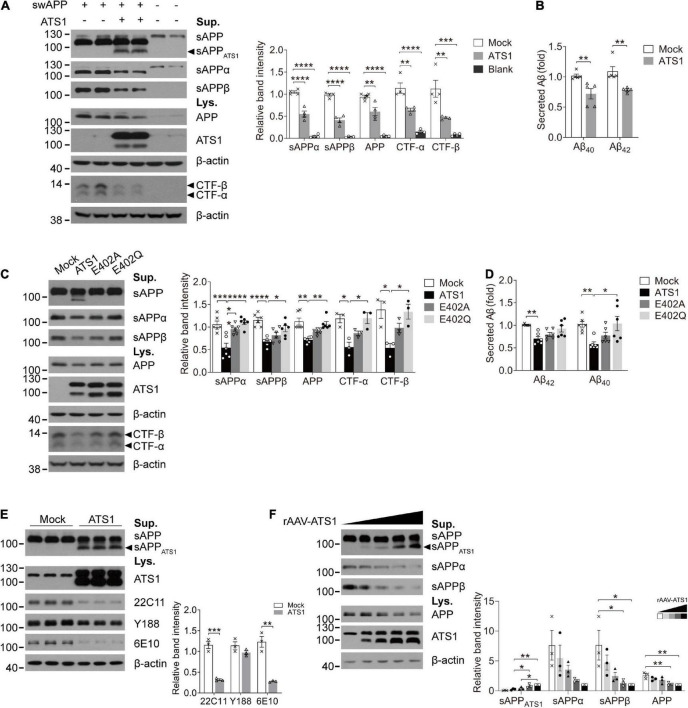
ATS1 contributed to APP metabolism and thereby decreased the generation of conventional products. **(A,B)** HEK 293T cells were co-transfected with *swAPP* and *ATS1* plasmids. **(A)** Western blot of APP pathway proteins (sAPP, sAPPα, sAPPβ, APP, and CTFs). A new fragment named sAPP_*ATS*1_ was observed and the relative band intensities indicated that the conventional cleavage of α and β-secretases was suppressed (*n* = 4, sAPPα, *P* < 0.0001, ATS1 vs. Mock; sAPPβ, *P* < 0.0001, ATS1 vs. Mock; APP, *P* < 0.01, ATS1 vs. Mock; CTF-α, *P* < 0.01, ATS1 vs. Mock; CTF-β, *P* < 0.01, ATS1 vs. Mock, one-way ANOVA). **(B)** ELISA quantification revealed a reduction in Aβ_40_ and Aβ_42_ in the conditioned medium (*n* = 5, Aβ_40_, *P* < 0.01, ATS1 vs. Mock; Aβ_42_, *P* < 0.01, ATS1 vs. Mock, Mann-Whitney *U*-test). **(C,D)** Two types of *ATS1* mutants with site-directed mutations at the active center and *swAPP* were transfected into HEK 293T cells. **(C)** Western blot of APP pathway proteins (sAPP, sAPPα, sAPPβ, APP, and CTFs). The new fragment sAPP_*ATS*1_ disappeared and the relative band intensities demonstrated that conventional products returned to control level in the E402Q system (*n* = 6, sAPPα, *P* < 0.001, ATS1 vs. Mock, *P* < 0.0001, E402Q vs. ATS1; sAPPβ, *P* < 0.0001, ATS1 vs. Mock, *P* < 0.05, E402Q vs. ATS1; APP, *P* < 0.01, ATS1 vs. Mock, *P* < 0.01, E402Q vs. ATS1; *n* = 3, CTF-α, *P* < 0.05, ATS1 vs. Mock, *P* < 0.05, E402Q vs. ATS1; CTF-β, *P* < 0.05, ATS1 vs. Mock, *P* < 0.05, E402Q vs. ATS1, one-way ANOVA or Kruskal-Wallis test). Specially, the CTFs were examined as verification and thus three replicates were conducted on Tris-tricine SDS-PAGE. **(D)** ELISA quantification showed the recovery of Aβ in E402Q conditioned medium when compared with ATS1 group (*n* = 6, Aβ_40_, *P* < 0.01, ATS1 vs. Mock, *P* < 0.05, E402Q vs. ATS1; Aβ_42_, *P* < 0.01, ATS1 vs. Mock, Kruskal-Wallis test). **(E)** Primary neurons were infected with rAAV*-ATS1* or Mock. Western blot was performed to analyze APP using three different antibodies. The relative band intensities showed that APP cleavage by ATS1 was identified by two antibodies (*n* = 3, 22C11, *P* < 0.001, ATS1 vs. Mock; 6E10, *P* < 0.01, ATS1 vs. Mock, Student’s *t*-test). **(F)** Primary neurons were infected with serially diluted rAAV*-ATS1*. Western blot showed that the contribution of ATS1 to APP cleavage strengthened with the concentration of rAAV*-ATS1* (*n* = 3, one-way ANOVA). Data were expressed as Mean ± SEM. *P < 0.05, **P < 0.01, ***P < 0.001, ****P < 0.0001. ATS1, ADAMTS1; Lys., lysates; Sup., supernatant; swAPP, human APP Swedish mutation; Mock, empty vector.

Previous studies have suggested that APP probably acts as an ECM constituent through the binding of its extracellular domain to ECM components, like heparin sulfate proteoglycans and collagen (Beher et al., 1996; Caceres and Brandan, 1997; van der Kant and Goldstein, 2015). Since *ADAMTS1* is implicated to hold associations with AD (Kunkle et al., 2019) and its product ADAMTS1 exhibits proteolytic activity (Shindo et al., 2000), we investigated whether ADAMTS1 participated in APP metabolism through co-transfecting HEK 293T cells with *swAPP* and *ADAMTS1*. Western blot data showed that the introduction of *ADAMTS1* resulted in a novel APP hydrolysate [about 75–85 kDa, soluble APP fragment cleaved by ADAMTS1 (sAPP_*ATS*1_)] accompanying traditionally recognized ones (Figure 4A). Moreover, ADAMTS1 expression caused an apparent decrease in sAPPs, CTFs and secreted Aβs simultaneously (Figures 4A,B, *P* < 0.01, compared with mock control). All these results demonstrated preliminarily the involvement of ADAMTS1 in APP proteolytic processing.

To verify the involvement, two types of site-directed mutations were introduced into the active center of ADAMTS1 (Alanine or Glutamine replacing Glutamate, E402A or E402Q) (Rodriguez-Manzaneque et al., 2002). Unsurprisingly, the mutants failed to generate sAPP_*ATS*1_ and conventional products returned to control level in the E402Q system (Figures 4C,D, *P* < 0.05, compared with ADAMTS1 group). Additionally, as shown in Figure 4E, consistent results were obtained from *ADAMTS1*-infected primary neurons, in which sAPP_*ATS*1_ appeared and conventional products showed clear attenuation (*P* < 0.01, compared with control group). Meanwhile, with the concentration of rAAV*-ADAMTS1*, the contribution on APP cleavage strengthened (Figure 4F).

**FIGURE 5:**
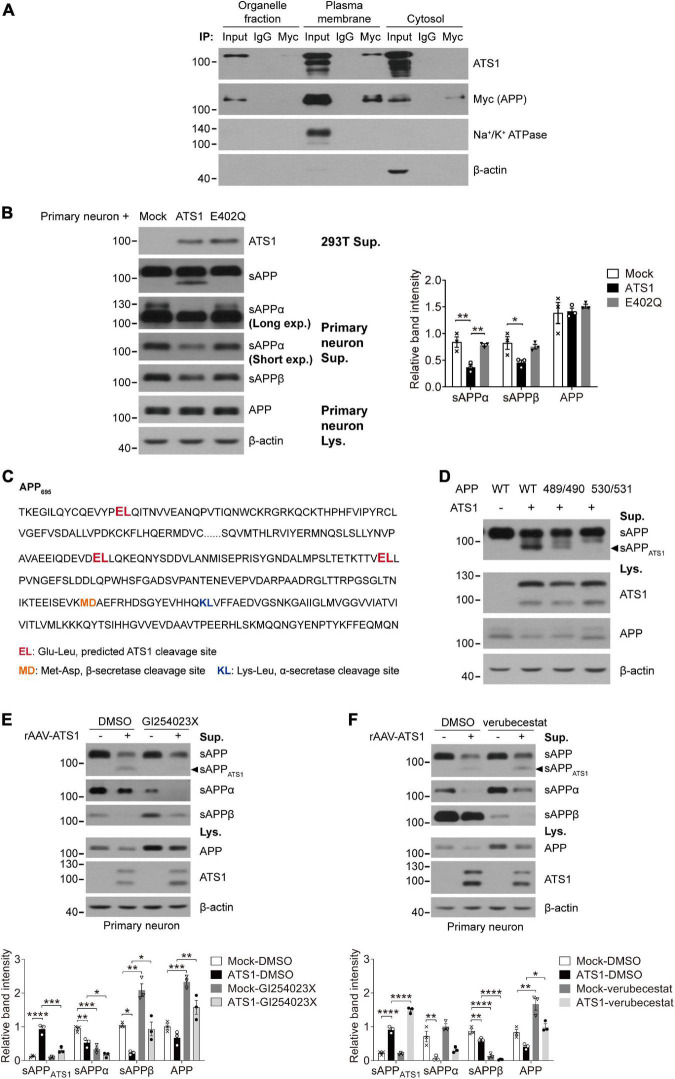
ATS1 hydrolyzed APP in plasma membrane at E530/L531 site and competed with BACE1. **(A)** HEK 293T cells transfected with *APP* and *ATS1* were subjected to cellular component separation and subsequent co-immunoprecipitation using Myc tag (APP) antibody. Western blot showed that the interaction of ATS1 and APP mainly occurred at the plasma membrane (labeled by Na^+^/K^+^ ATPase). **(B)** Primary neurons were treated with conditioned medium from HEK 293T cells transfected with mock, *ATS1* or *E402Q* mutant and subjected to western blot of APP pathway proteins sAPP, sAPPα, sAPPβ, and APP. The relative band intensities of cleavage products were shown. Primary neurons exposed to the supernatant of *ATS1*-transfected HEK 293T cell culture produced sAPP_*ATS*1_ and exhibited reduced levels of conventional products when compared with mock control or *E402Q*-transfected group (*n* = 3, sAPPα, *P* < 0.01, ATS1 vs. Mock, *P* < 0.01, E402Q vs. ATS1; sAPPβ, *P* < 0.05, ATS1 vs. Mock, one-way ANOVA). Moreover, long exposure time resulted in the detection of endogenous sAPPα of HEK 293T cells with lower protein level but higher molecular weight. **(C)** Predicted ATS1 cleavage sites were shown (emphasized in red), and the latter two sites E489/L490 and E530/L531 within amino acids 420–580 of APP_695_ were selected as the potential targets of ATS1. **(D)** HEK 293T cells were transfected with APP mutants holding A489/A490 or A530/A531. Western blot analysis showed that sAPP_*ATS*1_ was erased in A530/A531 group. **(E,F)** Primary neurons infected with rAAV*-ATS1* were treated with ADAM10 inhibitor GI254023X (20 μM) or BACE1 inhibitor verubecestat (1 μM) for 48 h. Western blot of APP pathway proteins (sAPP_*ATS*1_, sAPPα, sAPPβ, and APP) was shown. The relative band intensities of sAPP_*ATS*1_ revealed the potential competition between ATS1 and BACE1 [**(E)** *n* = 3, sAPP_*ATS*1_, *P* < 0.001, ATS1-GI254023X vs. ATS1-DMSO, one-way ANOVA; **(F)** *n* = 3, sAPP_*ATS*1_, *P* < 0.0001, ATS1-verubecestat vs. ATS1-DMSO, one-way ANOVA]. *P < 0.05, **P < 0.01, ***P < 0.001, ****P < 0.0001. Data were expressed as Mean ± SEM. ATS1, ADAMTS1; IP, immunoprecipitation; Lys., lysates; Sup., supernatant; Mock, empty vector; Long exp., long exposure time; Short exp., short exposure time; WT, wild type; 489/490, A489/A490; 530/531, A530/A531.

Next, we performed co-immunoprecipitation to further illustrate the interaction of APP and ADAMTS1. As shown in Supplementary Figure 3A, the interaction was confirmed reciprocally using ADAMTS1 (FLAG) and APP (Myc) antibodies. Specially, the positive result was only observed in the plasma membrane fraction (Figure 5A). Furthermore, when primary neurons were exposed to the supernatant of *ADAMTS1*-transfected HEK 293T cell culture, the appearance of sAPP_*ATS*1_ and the sharp decrease of conventional products (*P* < 0.05, compared with mock control) of primary neurons (clearly different from endogenous sAPPs of HEK 293T cells with higher molecular weight (Koch et al., 2012; Supplementary Figure 3B and Figure 5B) were still observed although the full-length APP showed no obvious variations (Figure 5B). This phenomenon did not happen when the supernatant was from cells containing the E402Q mutation (Figure 5B), suggesting that ADAMTS1 was a secretase.

It has been reported that ADAMTS1 cleaves the covalent bond between Glutamate and Leucine (Rodriguez-Manzaneque et al., 2002; Westling et al., 2002). We screened the APP sequence and found three Glu-Leu sites (Figure 5C, emphasized in red). As the molecular mass of sAPP_*ATS*1_ was about 75–85 kDa, the latter two Glu-Leu sites (E489/L490 and E530/L531, Figure 5C) within amino acids 420–580 of APP_695_ were selected as potential targets of ADAMTS1. By exposing APP_695_ mutants A489/A490 and A530/A531 to *ADAMTS1*-containing HEK 293T cells, the absence of sAPP_*ATS*1_ fragments in conditioned medium suggested that the cleavage site might be E530/L531 (Figure 5D). Additionally, the sAPP_*ATS*1_ fragments in A489/A490 also showed a decline, but full-length APP in cell lysate held the same level as WT control (Figure 5D). It was speculated that the mutation within E489/L490 might cause some modest structural modification of APP and thus partially limit ADAMTS1’s cleavage. However, further investigations should still be conducted using purified APP mutant and ADAMTS1 protein to verify the exact cleavage site.

While investigating protease inhibitors affecting ADAMTS1, we observed that the ADAM10 inhibitor GI254023X contributed to the downregulation of sAPP_*ATS*1_ (Supplementary Figure 3C and Figure 5E, *P* < 0.05, compared with DMSO group), and BACE1 inhibitor verubecestat resulted in accumulated sAPP_*ATS*1_ in HEK 293T cells and primary neurons (Supplementary Figure 3D and Figure 5F, *P* < 0.001, compared with DMSO group). Collectively, the data indicated that ADAMTS1 and BACE1 competed for the same substrate.

### The cognition of 5 × FAD mice was alleviated by hippocampal high-level *A Disintegrin and Metalloproteinase with Thrombospondin motifs 1* through the attenuation of amyloid load

**FIGURE 6:**
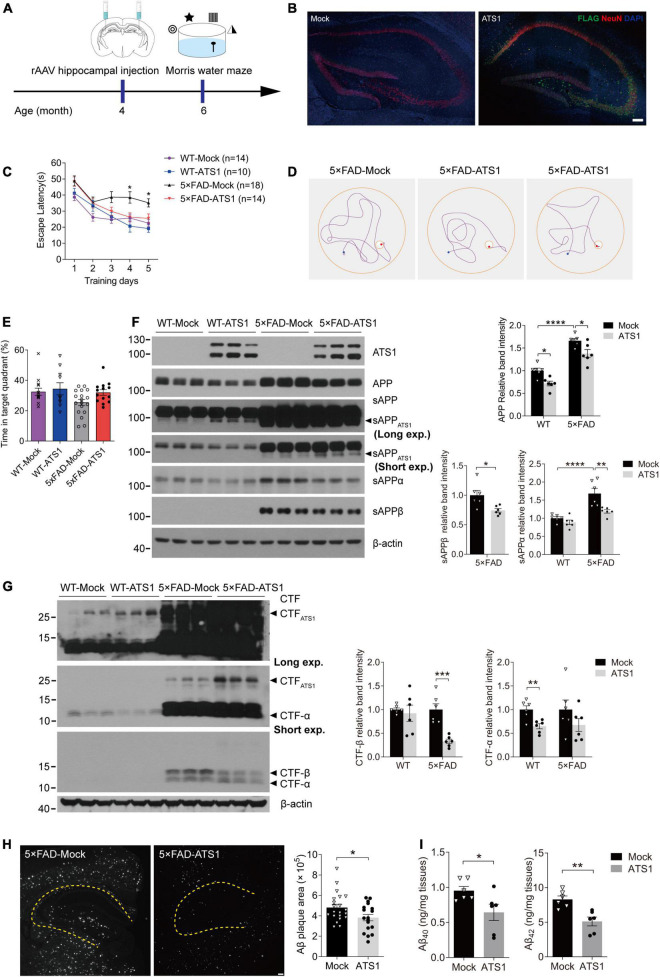
High-level ATS1 in hippocampus attenuated amyloid load of 5 × FAD mice and improved the cognitive behavior. **(A)** Scheme of rAAV injection and Morris water maze test performed on 5 × FAD mice. **(B)** Expression of rAAV*-ATS1-FLAG* and Mock. Nuclei (by DAPI), ATS1 (by FLAG), neuron (by NeuN) were labeled in hippocampus. Scale bars, 100 μm. (**C–E**) Morris water maze tests were performed on WT and 5 × FAD mice 2 months after injection of rAAV (*n* = 14 for WT-Mock, *n* = 10 for WT-ATS1, *n* = 18 for 5 × FAD-Mock and *n* = 14 for 5 × FAD-ATS1). **(C)** The escape latency was recorded during the 5-day training, showing that 5 × FAD mice with ATS1 spent less time during the learning trial when compared with mock control [DAY 4, *P* < 0.05, 5 × FAD-ATS1 vs. 5 × FAD-Mock; DAY 5, *P* < 0.05, 5 × FAD-ATS1 vs. 5 × FAD-Mock. Two-way repeated measures ANOVA, day × group *F*(3, 52) = 1.153, *P* > 0.05, no significant interaction; group *F*(3, 52) = 16.820, *P* < 0.0001]. **(D)** Representative navigation routes of 5 × FAD-Mock and 5 × FAD-ATS1 mice to the hidden platform during the last day of training. **(E)** The probe trial was performed 24 h after the last trial of hidden platform task, and the percentage of searching time in the target quadrant indicated that *ATS1*-transfected mice exhibited an apparent upward trend in the probability of finding the target quadrant (Kruskal-Wallis test). **(F)** Western blot of APP pathway proteins in WT and 5 × FAD mice infected with rAAV*-ATS1-FLAG* or Mock was shown. The relative band intensities showed reduced APP and sAPPβ levels in ATS1 group when compared with respective mock control and sAPPα also exhibited a downward trend (*n* = 6, APP in WT, *P* < 0.05, ATS1 vs. Mock; APP in 5 × FAD, *P* < 0.05, ATS1 vs. Mock; sAPPα in 5 × FAD, *P* < 0.01, ATS1 vs. Mock; sAPPβ in 5 × FAD, *P* < 0.05, ATS1 vs. Mock, one-way ANOVA or Student’s *t*-test). **(G)** Western blot of CTFs in WT and 5 × FAD mice infected with rAAV*-ATS1-FLAG* or Mock was shown. The relative band intensities revealed the accumulation of CTF_*ATS*1_ and reduced CTFs levels in ATS1 group when compared with respective mock control (*n* = 6, CTF-β in 5 × FAD, *P* < 0.001, ATS1 vs. Mock; CTF-α in WT, *P* < 0.01, ATS1 vs. Mock, Student’s *t*-test). **(H)** Immunofluorescence showing Aβ plaques in the hippocampus. Scale bar, 100 μm. Quantification of the surface area of Aβ plaques demonstrated the mitigated pathology by *ATS1* overexpression (*n* = 7 for 5 × FAD-Mock and *n* = 6 for 5 × FAD-ATS1, *P* < 0.05, ATS1 vs. Mock, Student’s *t*-test). **(I)** ELISA quantification of Aβ_40_ and Aβ_42_ in hippocampus lysates from 6-month-old mice certified the reduction of total Aβs caused by ATS1 (*n* = 6, Aβ_40_, *P* < 0.05, ATS1 vs. Mock; Aβ_42_, *P* < 0.01, ATS1 vs. Mock, Student’s *t*-test). *P < 0.05, **P < 0.01, ***P < 0.001, ****P < 0.0001. Data were expressed as Mean ± SEM. ATS1, ADAMTS1; WT, wild type; Long exp., long exposure time; Short exp., short exposure time.

Given that ADAMTS1 affected APP metabolism and reduced Aβ, we explored whether high-level ADAMTS1 improved the cognitive performance of 5 × FAD mice. To this end, the stereotaxic injection and subsequent Morris water maze test were performed (Figure 6A). The expression of *ADAMTS1-FLAG* was validated in Figure 6B. After 5 days of training, 5 × FAD mice without *ADAMTS1* still spent much more time (approximately 40 s) on locating the platform (Figures 6C,D, *P* < 0.05, compared with other groups) than those expressing *ADAMTS1*. Moreover, in the probe trial, *ADAMTS1*-transfected mice exhibited an apparent upward trend in the probability of finding the correct quadrant (Figure 6E). These results suggested that ADAMTS1 brought improvement to the cognitive functions of 5 × FAD mice.

Aβ-related proteins in 5 × FAD mice with and without *ADAMTS1* were examined to further explore the underlying mechanism of cognitive improvement. As shown in Figure 6F, consistent results were obtained from *in vivo* studies: sAPP_*ATS*1_ appeared and full-length APP showed clear downregulation. Moreover, the further check on conventional sAPPs and CTFs demonstrated that the β-cleavage in 5 × FAD mice significantly reduced and meanwhile APP hydrolysates resulted from α-secretase also exhibited downward trends (Figures 6F,G). Additionally, the western blot result of CTF_*ATS*1_ illustrated its accumulation which corresponded to that of sAPP_*ATS*1_ (Figure 6G).

Moreover, the amyloid burden was also mitigated by ADAMTS1 expression, which was revealed by the decreased Aβ-positive area in hippocampus expressing *ADAMTS1* (Figure 6H). In addition, a sharp reduction in Aβs was also certified by ELISA data (Figure 6I, compared with mock control), further confirming the effects of ADAMTS1 on Aβ lessening.

## Discussion

In this study, we first identified that a rare SNP c.–2067A > C of *ADAMTS1* potentially correlated with a reduced risk of AD, and that the novel variant and cognitive activities could significantly stimulate *ADAMTS1* expression. Further functional studies demonstrated that elevated ADAMTS1 transferred APP metabolism thus leading to reduced soluble Aβ generation. Moreover, the spatial cognition of AD mice was notably alleviated in the Morris water maze test through the reduction of Aβ plaques.

The progressive AD is now regarded as the leading cause of dementia and is partially driven by genetics. Previous GWAS has identified a large number of susceptibility loci for AD, like SNP “rs75932628” coding R47H substitution of TREM2 (Guerreiro et al., 2013; Jonsson et al., 2013). The R47H variant results in function-lost protein, although it possesses an extremely low MAF (Yeh et al., 2016; Song et al., 2017). Similar pattern seemed to be present here, that was, though the rare variant c.–2067A > C at *ADAMTS1* promoter had an MAF of 0.26%, it triggered enhanced fundamental transcription of *ADAMTS1*. However, given the limitation of sample size in identifying rare variants, the correlation of c.–2067A > C with AD still requires larger-scale cohort for further analysis.

In the past decade, nearly no monoclonal antibody targeting Aβ has succeeded in clinical trials, which raises the debates about Aβ’s central role in AD. However, the amyloid hypothesis refers to a continuous process lasting for 15–20 years before the onset of clinical symptoms (Rowe et al., 2010; Zhao et al., 2020), and thereby AD may begin much long before the so-called “early clinical stage” (Brookmeyer et al., 2018). This delay may lead to the pathogenic deposition of Aβ plaques in patients’ brains and consequent failure of antibody treatment. Considering the involvement of ADAMTS1 in APP metabolism, its relatively higher protein level resulted from the genetic variant or enriched cognitive activities might make carriers benefit during the early life.

Disrupting the process of APP cleavage to reduce Aβ generation represents an approach to delay AD. Based on this, BACE1 inhibitors have been developed. However, BACE1 has substrates other than APP, such as Jagged 1 (Hu et al., 2013), neuregulin 1 (Savonenko et al., 2008), and seizure-related protein 6 (Pigoni et al., 2016), and BACE1 cleavage is essential for their physiological functions. Thus, the clinical potential of current BACE1 inhibitors is largely limited due to numerous side effects through suppressing the cleavage of untargeted substrates. Therefore, competing with BACE1 for APP processing may serve as an attractive alternative. After ADAMTS1 cleavage at E530/L531, the CTF_*ATS*1_ (531–695 amino acids) still contained BACE1 site. However, the observed reduction of Aβ and CTF-β indicated that BACE1 could not further process CTF_*ATS*1_, suggesting that this fragment might not have accessible spatial structure for BACE1. On the other hand, previous studies have indicated that BACE1 primarily hydrolyzes APP within endosomes (Sannerud et al., 2011; Buggia-Prevot et al., 2013) and the internalization of APP from plasma membrane to endosomes is essential for BACE1 cleavage (Shahani et al., 2015; Yi et al., 2021). Considering the fractionation results that APP-ADAMTS1 interaction mainly occurred on plasma membranes, the binding of APP to ADAMTS1 might inhibit its transport and consequently block BACE1 cleavage.

When cells were treated with ADAM10 inhibitor, the decrease in sAPP_*ATS*1_ indicated limited cleavage of APP by ADAMTS1. Previous studies have shown that ADAMTS1 and ADAM10 own common tissue inhibitor TIMP-3 of which N-terminal residue blocks the active site of enzyme (Baker et al., 2002; Murphy and Nagase, 2008). Thus, the use of GI254023X might simultaneously inhibit ADAMTS1 while acting on ADAM10 cleavage. Additionally, ADAMTS1 and ADAM10 belong to ADAM family who has both disintegrin and zinc endopeptidases domains (Brocker et al., 2009), which implies that they may function on ECM and adhesion molecules (Greening et al., 2015; Shimoda et al., 2021) by similar approach. Therefore, these two enzymes might compete resulting in reduced α-cleavage in ADAMTS1-overexpressed condition.

Actually, cognitive functions are affected by multiple factors including aging, education, and cognitive activities (Phillips, 2017; Yu et al., 2020). Numerous studies focusing on cognitive reserve have shown the retardant beneficial effects of an active lifestyle on cognitive decline in the elderly (Wilson et al., 2002, 2007). Recent clinical data have also repeatedly emphasized that frequent stimuli on cognition of the elderly may delay the onset of AD by as long as 5 years (Wilson et al., 2021). However, the underlying mechanism through which cognitive activities and cognitive functions are bridged remains unclear. As to experimental mice, cognitive activities can be performed in the so-called EE where mice receive physical, cognitive, and social stimulation. We found that ADAMTS1 was clearly upregulated by EE-housing, which verified the speculation that *ADAMTS1* was induced as a result of neuronal activities involved in cognitive events. Moreover, our findings demonstrated that the promoter activity of *ADAMTS1* was clearly induced by IEG zif268. This result suggested the correlation between gene expression and IEG-associated stimulus. Accumulated studies have also reported that zif268 expression is sensitive to specific learning paradigms such as spatial memory, object recognition memory and so on. Deficiency of zif268 severely damages the cognitive performance in different behavioral tests (Gallo et al., 2018). Moreover, it is reported that a mouse model suffering from neurodegenerative disease in long-term EE shows notably attenuated progression and ameliorated amyloid load (Lazarov et al., 2005; Hu et al., 2010). Considering all the above results, we speculated that zif268 played a key role in the bridge of cognitive activities and *ADAMTS1* expression, and in this way delayed the disease progression.

In summary, based on the association of *ADAMTS1* with AD implied by GWAS, our work illuminated for the first time the significant role of ADAMTS1 as an APP hydrolase that connected both genetic susceptibility and acquired activities with amyloid processing, which might be instructive on further understanding the complex pathogenesis of AD.

## Data availability statement

The original contributions presented in the study are included in the article/Supplementary material, further inquiries can be directed to the corresponding author/s.

## Ethics statement

The studies involving human participants were reviewed and approved by the Institutional Review Board of Chinese Academy of Medical Sciences and Peking Union Medical College. The patients/participants provided their written informed consent to participate in this study. The animal study was reviewed and approved by the Institutional Review Board of Chinese Academy of Medical Sciences and Peking Union Medical College.

## Author contributions

QX and LS designed the research. YQ, XZ, and GL performed the experiments. WZ performed statistics. YQ wrote the manuscript. All authors contributed to the article and approved the submitted version.
