# Supplementary material for: Induction of A Disintegrin and Metalloproteinase with Thrombospondin motifs 1 by a rare variant or cognitive activities reduces hippocampal amyloid-β and consequent Alzheimer’s disease risk
Source: Front Aging Neurosci. 2022 Aug 9;14:896522. doi: 10.3389/fnagi.2022.896522 (PMC9395645; doi:10.3389/fnagi.2022.896522)
Supplement: Supplementary file 1 [file Data_Sheet_1.docx]

Supplementary Material

## Supplementary Figures


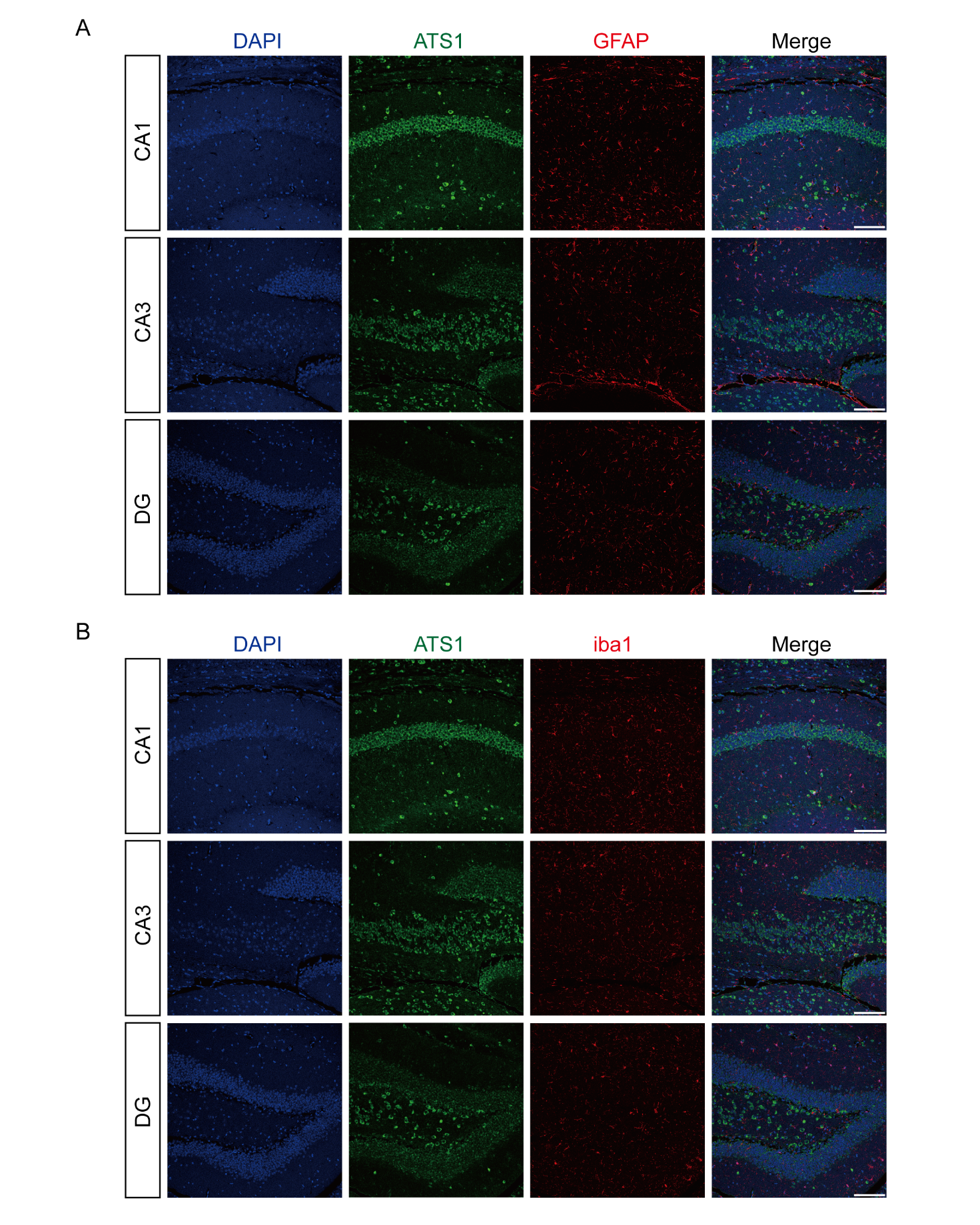


**Supplementary Figure S1.** ATS1 was rarely expressed by astrocytes or microglia. **(A, B)** Nuclei (by DAPI), ATS1 (by antibody), astrocytes (by GFAP) and microglia (by iba1) were labeled in CA1, CA3 and DG regions. Scale bars, 100 μm. ATS1, ADAMTS1; DG, dentate gyrus.


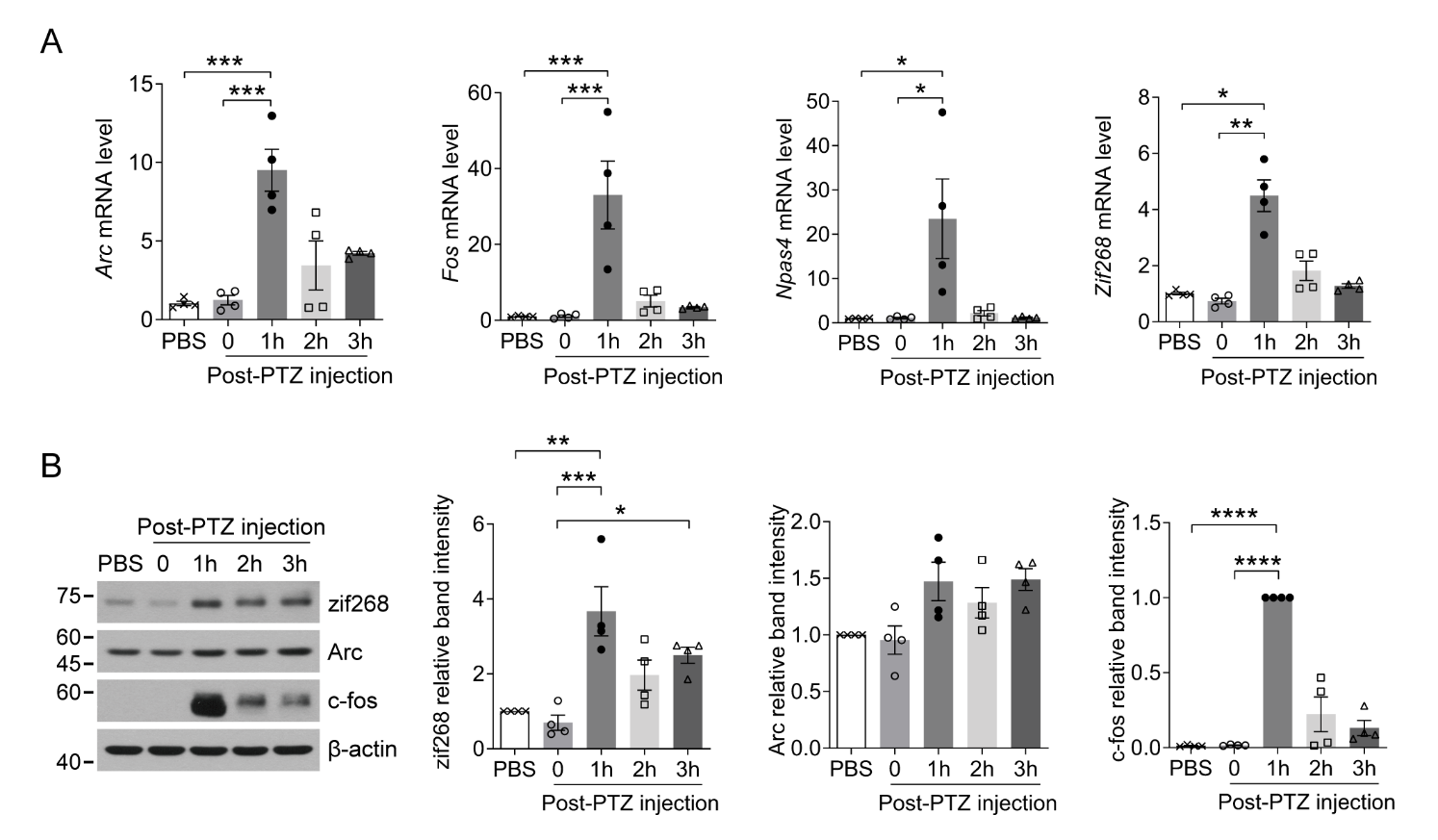


**Supplementary Figure S2.** 10-week-old WT male mice were intraperitoneally injected with PTZ to induce synaptic activation and sacrificed at different timepoints. Immediate early genes *Arc*, *Fos*, *Npas4* and *Zif268* were subsequently induced by the activation. **(A)** The relative mRNA levels of *Arc*, *Fos*, *Npas4* and *Zif268* were significantly upregulated 1 h post injection (*n* = 4; *Arc*, *P* < 0.001, 1 h vs PBS, *P* < 0.001, 1 h vs 0; *Fos*, *P* < 0.001, 1 h vs PBS, *P* < 0.001, 1 h vs 0; *Npas4*, *P* < 0.05, 1 h vs PBS, *P* < 0.05, 1 h vs 0; *Zif268*, *P* < 0.05, 1 h vs PBS, *P* < 0.01, 1 h vs 0, one-way ANOVA or Kruskal-Wallis test). **(B)** Western blot of zif268, Arc and c-fos at different timepoints. The relative band intensities showed that zif268 and c-fos were upregulated 1 h post injection (*n* = 4; zif268, *P* < 0.01, 1 h vs PBS, *P* < 0.001, 1 h vs 0, *P* < 0.05, 3 h vs 0; c-fos, *P* < 0.0001, 1 h vs PBS, *P* < 0.0001, 1 h vs 0, one-way ANOVA). **P* < 0.05, ***P* < 0.01, ****P* < 0.001, *****P* < 0.0001. Data was expressed as Mean ± SEM. PTZ, pentylenetetrazol; WT, wild type.


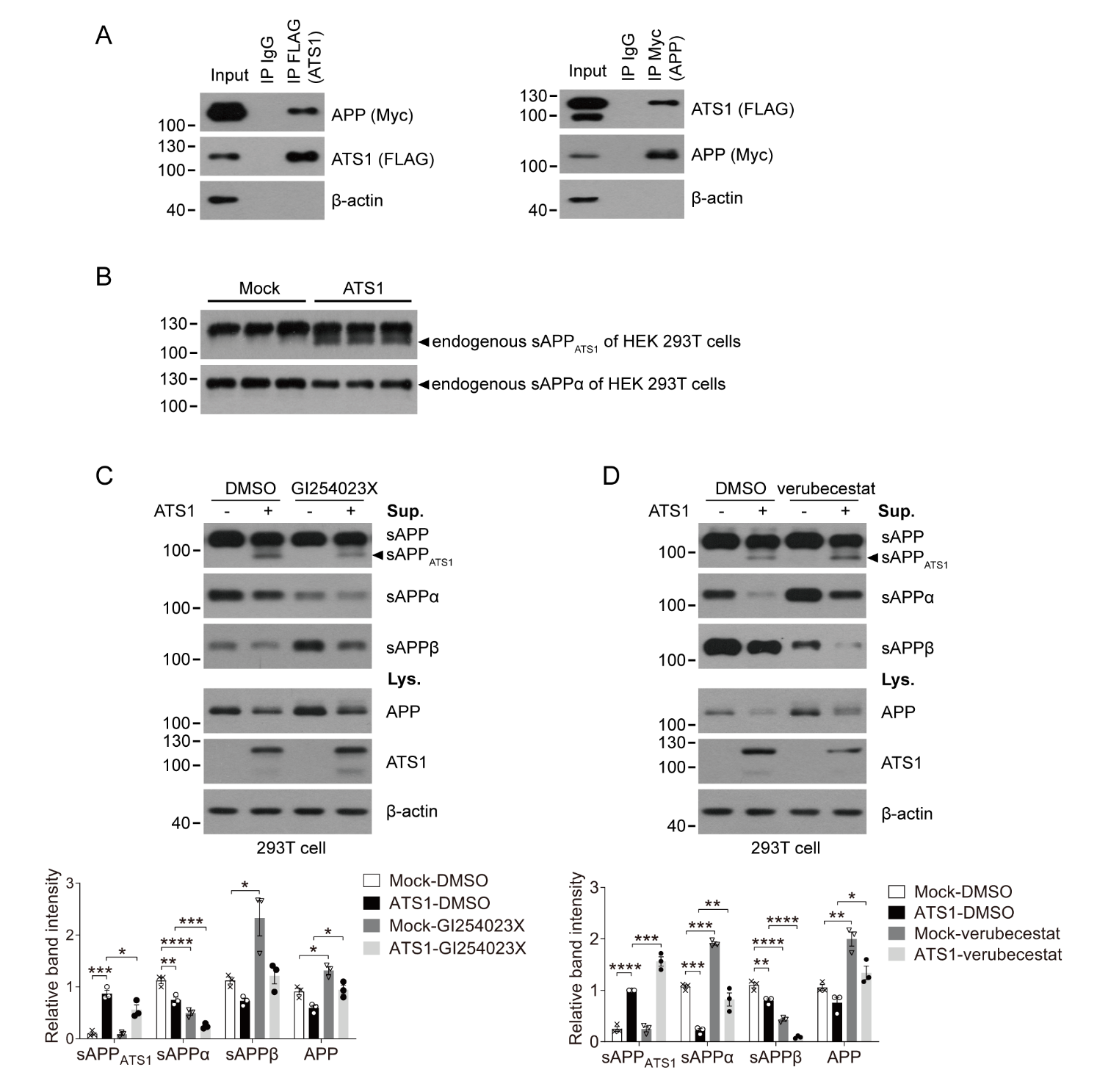


**Supplementary Figure S3.** The interaction of ATS1 and APP was blocked by the inhibitor of α-secretase while promoted by that of β-secretase. **(A)** Co-immunoprecipitation of cell lysates from HEK 293T cells transfected with *APP* and *ATS1*. Western blot showed the interaction of APP and ATS1 with both ATS1 and APP antibodies. **(B)** Western blot of soluble APP metabolites in HEK 293T cells merely transfected with *ATS1* or mock showed the molecular weight of endogenous sAPP_ATS1_ and sAPPα which was clearly distinguished from that of products derived from primary neurons. **(C, D)** HEK 293T cells transfected with *APP* and *ATS1* were treated with ADAM10 inhibitor GI254023X (20 μM) or BACE1 inhibitor verubecestat (1 μM) for 24 h. Western blot of APP pathway proteins (sAPP_ATS1_, sAPPα, sAPPβ and APP) was shown. The relative band intensities indicated that ATS1 activity was suppressed by **(C)** GI254023X (*n* = 3, sAPP_ATS1_, *P* < 0.05, ATS1-GI254023X vs ATS1-DMSO, one-way ANOVA) while promoted by **(D)** verubecestat (*n* = 3, sAPP_ATS1_, *P* < 0.001, ATS1-verubecestat vs ATS1-DMSO, one-way ANOVA). **P* < 0.05, ***P* < 0.01, ****P* < 0.001, *****P* < 0.0001. ATS1, ADAMTS1; IP, immunoprecipitation; Lys., lysates; Sup., supernatant. Mock, empty vector.
